# An Overview of the Synthesis and Antimicrobial, Antiprotozoal, and Antitumor Activity of Thiazole and Bisthiazole Derivatives

**DOI:** 10.3390/molecules26030624

**Published:** 2021-01-25

**Authors:** Anca-Maria Borcea, Ioana Ionuț, Ovidiu Crișan, Ovidiu Oniga

**Affiliations:** 1 Department of Pharmaceutical Chemistry, “Iuliu Hațieganu” University of Medicine and Pharmacy, 41 Victor Babeș Street, 400012 Cluj-Napoca, Romania; borcea.anca@umfcluj.ro (A.-M.B.); onigao65@yahoo.com (O.O.); 2 Preclinic Department, Pharmacy Specialization, Faculty of Medicine, Lucian Blaga University of Sibiu, 2A Lucian Blaga Street, 550169 Sibiu, Romania; 3 Department of Organic Chemistry, “Iuliu Hațieganu” University of Medicine and Pharmacy, 41 Victor Babeș Street, 400012 Cluj-Napoca, Romania; ocrisan@umfcluj.ro

**Keywords:** thiazole, bisthiazole, synthesis, derivatives, biological activity

## Abstract

Thiazole, a five-membered heteroaromatic ring, is an important scaffold of a large number of synthetic compounds. Its diverse pharmacological activity is reflected in many clinically approved thiazole-containing molecules, with an extensive range of biological activities, such as antibacterial, antifungal, antiviral, antihelmintic, antitumor, and anti-inflammatory effects. Due to its significance in the field of medicinal chemistry, numerous biologically active thiazole and bisthiazole derivatives have been reported in the scientific literature. The current review provides an overview of different methods for the synthesis of thiazole and bisthiazole derivatives and describes various compounds bearing a thiazole and bisthiazole moiety possessing antibacterial, antifungal, antiprotozoal, and antitumor activity, encouraging further research on the discovery of thiazole-containing drugs.

## 1. Introduction

Nitrogen-containing heterocyclic compounds play an important role in the drug discovery process, as approximately 75% of FDA (Food and Drug Administration)-approved small-molecule drugs contain one or more nitrogen-based heterocycles [1]. Thiazole, or 1,3-thiazole, belongs to the class of azoles and contains one sulfur atom and one nitrogen atom at positions 1 and 3. Its diverse biological activity is reflected in a large number of clinically approved thiazole-containing compounds with an extensive range of pharmacological activities. Most of these compounds are 2,4-disubstituted thiazole derivatives, and only a few are 2,5-disubstituted or 2,4,5-trisubstituted thiazoles [2]. 

**Figure 1:**
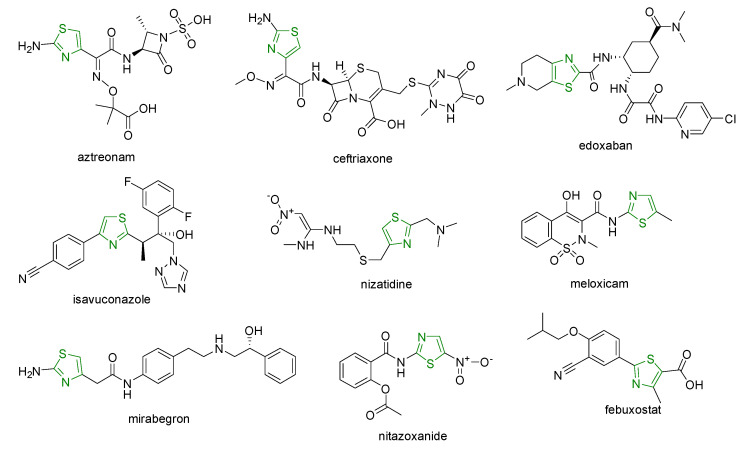
Clinical drugs bearing one thiazole ring.

Several drugs such as sulfathiazole; aztreonam; numerous cephems (ceftaroline, cefotiam, ceftibuten, cefixime, ceftriaxone, cefotaxime, ceftazidime, cefmenoxime, ceftizoxime, cefepime, cefdinir) with antibacterial effects; pramipexole with antiparkinsonian activity; edoxaban with antithrombotic effects; isavuconazole with antifungal effects; famotidine and nizatidine with antiulcer activity; meloxicam with anti-inflammatory effects; tiazofurin, dabrafenib, dasatinib, ixabepilone, and epothilone with antitumor effects; mirabegron as a β3-adrenergic receptor agonist; nitazoxanide and thiabendazole with antiparasitic effects; and febuxostat with antigout activity contain one thiazole moiety in the structure (Figure 1) [2,3,4].

**Figure 2:**
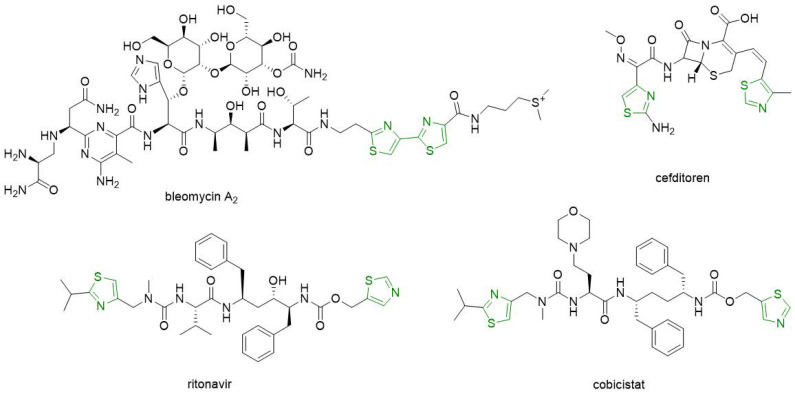
Clinical drugs bearing two thiazole rings.

Compounds bearing two thiazole rings, such as the antitumor drug bleomycin, the antiretroviral agent ritonavir, the pharmacokinetic enhancer for HIV drugs cobicistat, and the antibacterial agent cefditoren, have been authorized on the pharmaceutical market (Figure 2) [2,5]. 

The high medicinal significance of this scaffold has attracted considerable attention from many researchers and encouraged the design and synthesis of numerous thiazole- and bisthiazole-containing compounds with diverse pharmacological activities, such as antibacterial [6], antifungal [7], antiprotozoal [8], antiviral [9,10], anticancer [11], anti-inflammatory [12,13,14,15,16], antioxidant [17], analgesic [18], anticonvulsant [19], antidiabetic [20], and antihypertensive [21] activities. Furthermore, thiazole compounds exhibiting a promising biological potential for the treatment of Alzheimer’s disease [22,23] and metabolic syndrome [24] have been reported in the scientific literature.

Nowadays, research in the field of antimicrobial drug design is focused on the discovery of novel targets and chemical entities that possess antibacterial activity in order to overcome the rapid development of drug resistance. The few antibiotics that have been recently approved are structurally related to older drugs, being susceptible to the same mechanisms of resistance [25]. Tropical diseases such as Chagas disease, leishmaniasis, and malaria occur especially in underdeveloped countries, around 266 million cases being reported globally [26]. At the present time, only a small number of drugs are available on the pharmaceutical market for the treatment of protozoan infections [27]. Cancer remains one of the leading causes of mortality worldwide. The lack of selectivity and target specificity, as well as the presence of toxicity and resistance, is an inadequate feature of the currently available anticancer drug therapy [3]. Considering all the above, there is an urgent need for continuous progress in the design and development of anti-infective and antitumor agents.

The current review systematically presents different methods for the synthesis of thiazole and bisthiazole derivatives. Moreover, various reports on the antimicrobial, antiprotozoal, and antitumor activities of thiazoles and bisthiazoles, mostly published within the past 10 years, are discussed, providing reference for further research regarding the development of new biologically active chemical entities.

## 2. Chemistry of Thiazole

**Figure 3:**
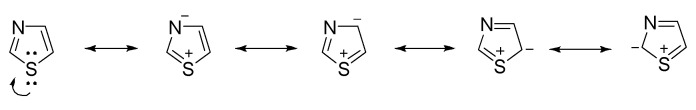
The resonance structures of thiazole.

Free thiazole is a pale-yellow flammable liquid, with a pyridine-like odor and a boiling point in the range of 116–118 °C. It has an aromatic character, due to the delocalization of a lone pair of electrons from the sulfur atom, resulting in a 6π-electron system. Also, its high aromaticity is highlighted by proton nuclear magnetic resonance, the chemical shift values of each proton within the thiazole ring being situated between 7.27 and 8.77 ppm. The resonance structures of thiazole are illustrated in Figure 3 [28,29].

**Figure 4:**
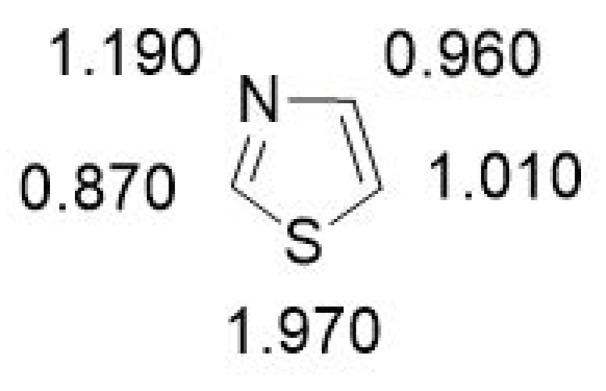
Calculated π-electron density of thiazole.

The calculated π-electron density revealed that the electrophilic substitution takes place preferentially at the C-5 position, followed by the C4-position (Figure 4). The nucleophilic substitution occurs at the C-2 position [30].

The acidity given by the presence of the three hydrogen atoms decreases in the order H2 >> H5 > H4 [4]. 

## 3. Synthesis of Thiazole Derivatives

Hantzsch synthesis is the oldest and most widely known method for the synthesis of a thiazole ring. The method consists of a cyclization reaction between alpha-halocarbonyl compounds and various reactants containing the N-C-S fragment. Examples of such compounds include thiourea, thioamides, thiosemicarbazides, and thiosemicarbazones [31]. 

**Scheme 1:**
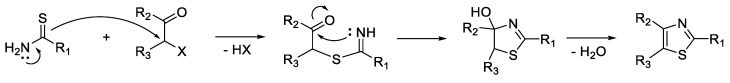
Reaction mechanism of Hantzsch thiazole synthesis.

The condensation of thioamides with various alpha-halocarbonyl compounds is commonly used. Many thiazoles with alkyl, aryl, or heteroaryl substituents at position 2, 4, or 5 can be obtained through this reaction. The reaction mechanism consists of the nucleophilic attack of the thioamide sulfur atom on the alpha carbon of the alpha-halocarbonyl, with the formation of an intermediate compound, which by subsequent dehydration leads to the corresponding thiazole (Scheme 1).

By the reaction of thiourea with alpha-halocarbonyl compounds, monosubstituted or disubstituted 2-aminothiazoles can be obtained, while by using other compounds containing thioamide moieties, such as thiosemicarbazides and thiosemicarbazones, 2-hydrazinothiazole and thiazol-2-yl-hydrazone derivatives can be synthesized in good yields. The condensation reactions occur through imino thioether and hydroxythiazoline intermediates, which are sometimes stable and isolable. The alpha-halocarbonyl component may be represented by alpha-haloketones and alpha-haloesters [32,33].

**Scheme 2:**
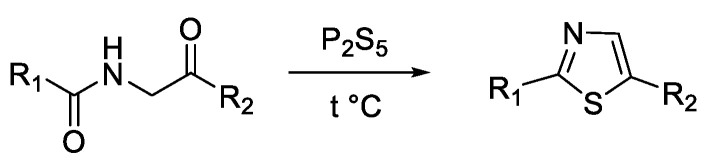
Gabriel thiazole synthesis.

Another method of obtaining thiazoles is represented by Gabriel synthesis, which consists of the cyclization reaction of acylaminocarbonyl compounds and a stoichiometric amount of phosphorus pentasulfide at 170 °C (Scheme 2) [28,34].

**Scheme 3:**
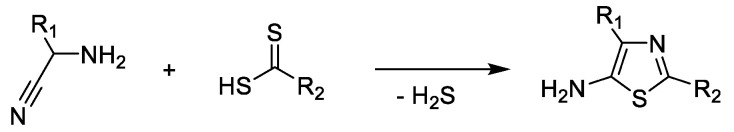
Cook–Heilbron synthesis of 5-aminothiazoles.

**Scheme 4:**
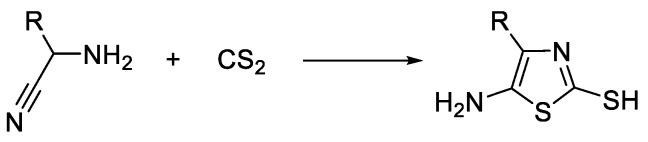
Cook–Heilbron synthesis of 5-amino-2-mercaptothiazoles.

Cook–Heilbron synthesis leads to 2,4-disubstituted 5-aminothiazole derivatives by the reaction of an α-aminonitrile and dithioacids or esters of dithioacids, carbon disulfide, carbon oxysulfide, or isothiocyanates under mild reaction conditions (Scheme 3). When carbon disulfide is used in the reaction, 5-amino-2-mercaptothiazole compounds are formed (Scheme 4) [32,33].

**Scheme 5:**
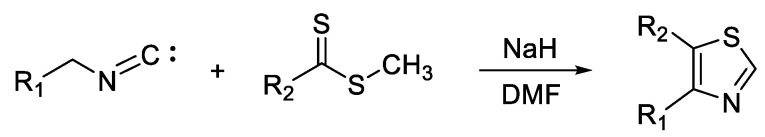
Synthesis of thiazoles from isocyanide derivatives and carboxymethyl dithioates.

Lingaraju et al. [35] efficiently synthesized a series of 4,5-disubstituted thiazole derivatives from active methylene isocyanides and methyl carbodithioates (Scheme 5). The reaction took place in the presence of a strong base, sodium hydride, and dimethylformamide (DMF) as a solvent. The short reaction time (10–30 min) and obtaining sufficiently pure final products in good yields are the main advantages of this method. Moreover, this procedure made it possible to obtain 2-unsubstituted thiazoles, which cannot be easily synthesized through other approaches such as Hantzsch or Cook–Heilbron synthesis.

**Scheme 6:**
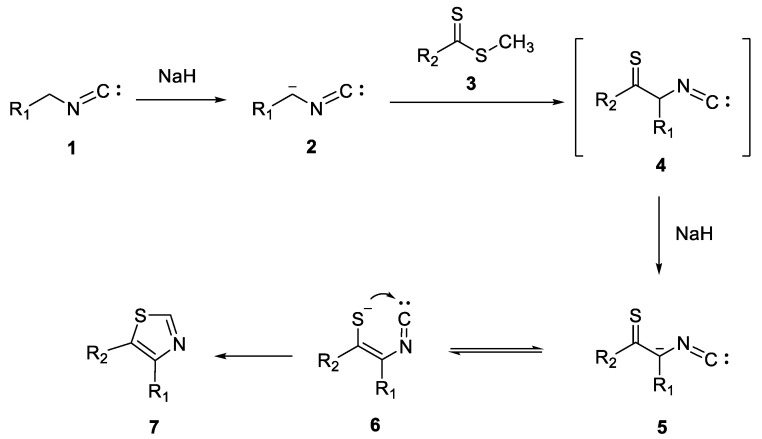
The proposed mechanism for the synthesis of 4,5-disubtituted thiazole derivatives.

The proposed mechanism is illustrated in Scheme 6. Carbanion **2**, formed by removing a proton from the active methylene group of compound **1,** in the presence of sodium hydride, attacks dithioester **3**, generating an unstable intermediate **4**. Subsequently, in the presence of sodium hydride, carbanion **5** is obtained, being in equilibrium with the enethiolate anion **6**. In the end, thiazole nucleus **7** is formed following the intramolecular cyclization of compound **6**.

**Scheme 7:**
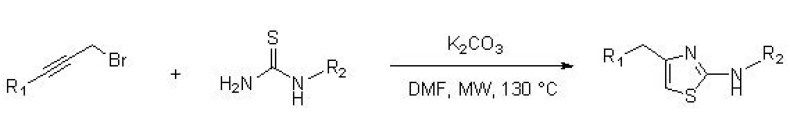
Synthesis of 2-aminothiazoles from substituted propargyl bromide and thioureas.

Castagnolo et al. [36] reported the synthesis of 2-aminothiazoles by a domino alkylation-cyclization reaction starting from various substituted propargyl bromides and thiourea derivatives. The synthesis was carried out in the presence of a stoichiometric amount of potassium carbonate and DMF as a solvent, at a temperature of 130 °C, under microwave irradiation, for 10 min (two cycles of 5 min each), as illustrated in Scheme 7.

This method is attractive due to the high availability of alkynes, which are an important alternative to α-haloketones used in Hantzsch synthesis, the main disadvantage of the latter being the strongly irritating character.

**Scheme 8:**
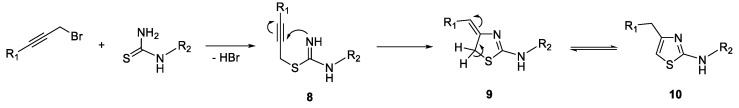
The proposed mechanism for the synthesis of 2-aminothiazole derivatives.

The proposed reaction mechanism is shown in Scheme 8. The initial step consists of the alkylation of thiourea or substituted thioureas, leading to the formation of intermediate **8**. Subsequently, compound **8** undergoes a 5-*exo*-dig cyclization reaction with the generation of intermediate **9**, which is transformed through an isomerization reaction into the final reaction product, 2-aminothiazole **10**.

**Scheme 9:**
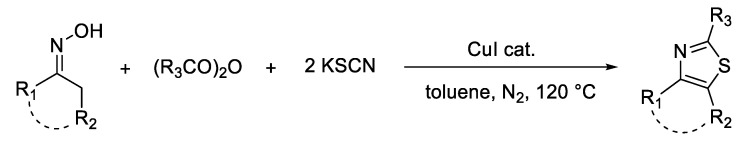
Synthesis of thiazoles from oximes, anhydrides, and KSCN.

Tang et al. [37] developed a method of obtaining thiazoles by a copper-catalyzed cyclization reaction starting from oximes, anhydrides, and potassium thiocyanate (Scheme 9). To optimize reaction conditions, the authors examined various solvents, such as 1,2-dichloroethane, 1,4-dioxane, acetonitrile and toluene, different copper salts (such as CuI, CuCl, CuBr, CuBr_2_, Cu(OAc)_2_, and Cu(OTf)_2_ and their replacement with other metal salts such as Fe(OAc)_2_, FeBr_2_, FeBr_3_, FeCl_3_, PdCl_2_, and AgCl), and different sulfur sources such as KSCN, NaSCN, NH_4_SCN, S, and Na_2_S.

Good to excellent yields (up to 85%) were obtained when toluene was used as a solvent, copper(I) iodide as a catalyst, and two equivalents of KSCN as a sulfur source. The reaction took place at a temperature of 120 °C, under a nitrogen atmosphere, for 24 h.

**Scheme 10:**
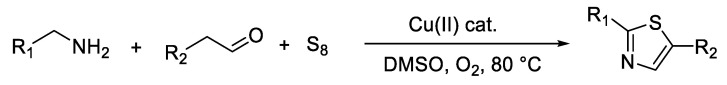
Synthesis of thiazoles from aldehydes, amines, and element sulfur.

Wang et al. [38] obtained a series of thiazole derivatives starting from aldehydes, amines, and element sulfur through an oxidation reaction catalyzed by copper salts in the presence of molecular oxygen (Scheme 10). The main advantages of this method are the high availability of the starting materials, the low-cost catalyst, and the use of a green oxidant.

**Scheme 11:**
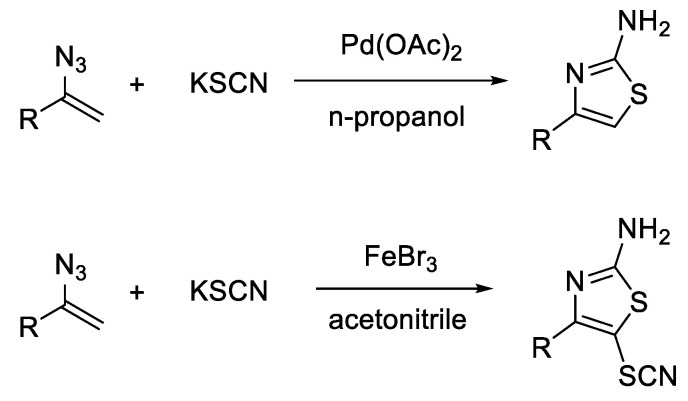
Synthesis of thiazoles from vinyl azides and potassium thiocyanate under different reaction conditions.

Chen et al. [39] successfully synthesized 4-substituted 2-aminothiazoles and 4-substituted 5-thiocyano-2-aminothiazoles starting from substituted vinyl azides and potassium thiocyanate under different reaction conditions. They found that the use of palladium(II) acetate as a catalyst and *n*-propanol as a solvent led to 4-substituted 2-aminothiazoles, after a reaction time of 12 h, at 80 °C, while in the presence of iron(III) bromide as a catalyst and acetonitrile as a solvent, 4-substituted 5-thiocyano-2-aminothiazoles were formed (Scheme 11).

When palladium(II) acetate was used as a catalyst, vinyl azides could be substituted with either aromatic (phenyl, naphthyl) or alkyl substituents, while the reaction catalyzed by iron(III) bromide took place only when vinyl azides were substituted with aromatic residues. In both cases, if the phenyl residue is substituted with an electron-withdrawing group (-NO_2_, -COOCH_3_), the reaction yield decreases. Moreover, if an electron-withdrawing group, such as an ester or a benzoyl group, binds directly to the alpha-carbon of vinyl azide, the reaction no longer takes place.

The study of the mechanism showed that the reaction occurred through an ionic mechanism, in the presence of palladium(II) acetate, and through a radical mechanism, in the presence of iron(III) bromide, thus explaining the formation of different reaction products [39].

Numerous heterocyclic compounds possessing the thiazole ring have been obtained in good to excellent yields using microwave irradiation [40,41]. Compared to conventional methods, microwave-assisted synthesis has several advantages, being an environmentally friendly and cost-effective tool, that leads to improved yields in short reaction times [42].

**Scheme 12:**
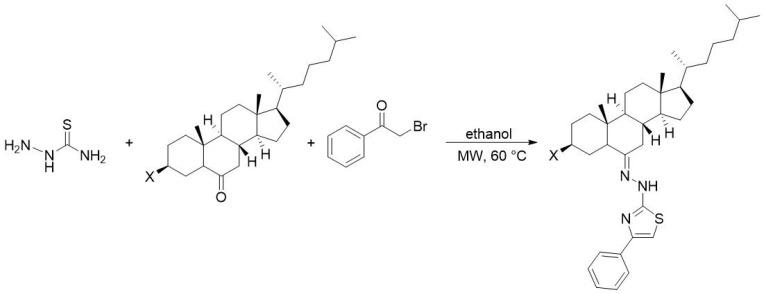
Synthesis of thiazoles from thiosemicarbazide, ketones, and 2-bromoacetophenone under microwave irradiation.

Asif et al. [43] developed a simple, one-pot, efficient method for the synthesis of novel steroidal thiazole derivatives through the condensation reaction of 2-bromoacetophenone, thiosemicarbazide, and steroidal carbonyl compounds (Scheme 12). The reaction took place under microwave heating at 60 °C, in ethanol, for 35–45 min, obtaining the target compounds in good yields (80–85%).

**Scheme 13:**
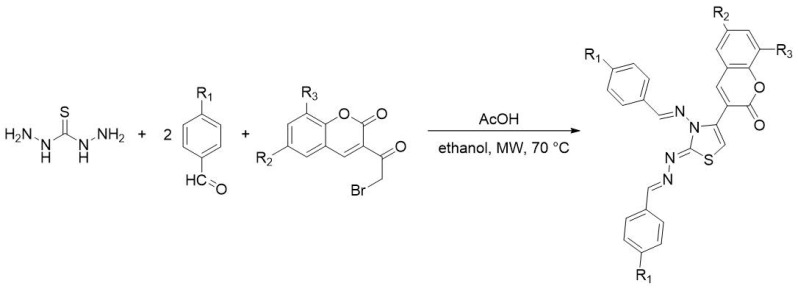
Synthesis of thiazoles from thiocarbohydrazide, aldehydes, and α-bromoketones under microwave irradiation.

Mamidala et al. [44] reported the synthesis of new coumarin-based thiazole derivatives, starting from thiocarbohydrazide, aldehydes, and α-halocarbonyl coumarins in a molar ratio of 1:2:1, under microwave heating (Scheme 13). Various solvents like methanol, ethanol, dimethyl sulfoxide (DMSO), and acetonitrile and different catalysts such as acetic acid, sulfuric acid, and hydrochloric acid were investigated in order to optimize the reaction regarding time and yield. Thus, it was observed that using ethanol as a solvent and a catalytic amount of acetic acid under microwave irradiation (70 °C, 210 W) led to high yields (88–93%) and short reaction times (5–8 min).

**Scheme 14:**
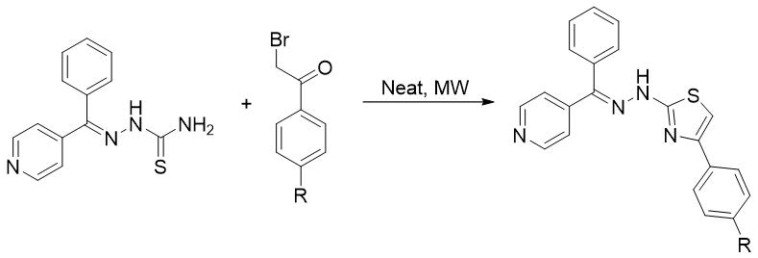
Synthesis of thiazoles from thiosemicarbazones and α-bromoketones under microwave irradiation.

Chinnaraja and Rajalakshmi [45] reported the microwave-assisted synthesis of novel hydrazinyl thiazole derivatives within 30–175 s under solvent- and catalyst-free conditions. Starting from either various carbonyl compounds, thiosemicarbazide, and alpha-haloketones or substituted thiosemicarbazones and alpha-haloketones (Scheme 14), the target compounds were obtained in good to excellent yield and high purity through an eco-friendly, one-pot procedure.

## 4. Biological Activity of Thiazole Derivatives

Among heterocyclic compounds, thiazole has proved its biological significance in medicinal chemistry, being a valuable scaffold in the design and synthesis of new drugs.

### 4.1. Antimicrobial Activity

Currently, research in the area of antimicrobial drug design and development has been stimulated due to the expansion of the bacterial resistance phenomenon, with the emergence of multidrug-resistant strains such as *Staphylococcus aureus*, *Enterococcus* sp., *Acinetobacter baumannii, Pseudomonas aeruginosa* and *Enterobacteriaceae* [46], and *Candida* sp. with intrinsic and acquired resistance to fluconazole [47].

Nowadays, particular attention is given to hybrid molecules containing a thiazole nucleus in combination with other pharmacophore features. Numerous hybrid molecules of thiazole combined with different heterocycles, such as thiophene, pyrazole, triazole, 1,3,4-oxadiazole, pyridine, 1,4-dihydropyridine, indole, quinoline, pyrimidine, pyrazine, triazine, and pyrazoline, were designed, synthesized, and evaluated as antimicrobial agents [48,49,50]. Moreover, novel hydrazones and Schiff bases bearing a thiazole scaffold have demonstrated good antimicrobial activity [51,52].

**Figure 5:**
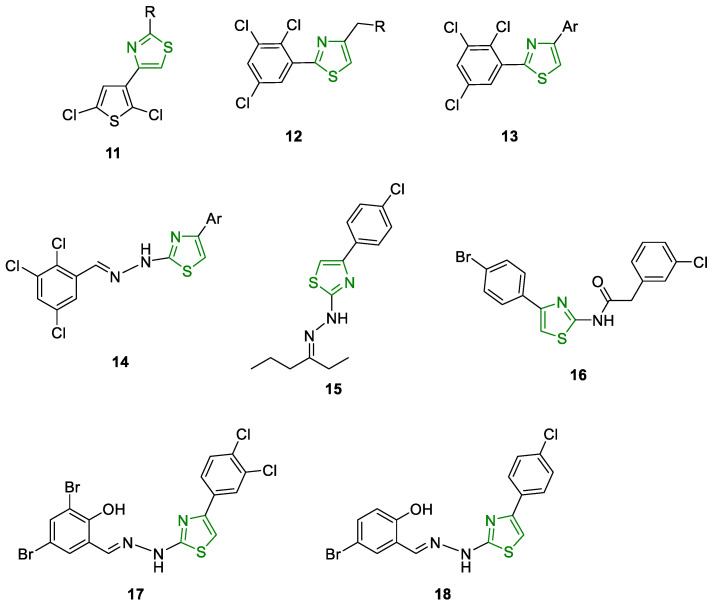
Thiazole derivatives with antimicrobial activity.

A new series of 2,5-dichloro thienyl-substituted thiazoles were efficiently obtained by Sarojini et al. [53] (compound **11**, Figure 5) and their minimum inhibitory concentration (MIC) values against four fungal strains (*Aspergillus fumigatus, Aspergillus flavus, Penicillium marneffei*, and *Trichophyton mentagrophytes*) and four bacterial strains (*Staphylococcus aureus, Escherichia coli, Klebsiella pneumoniae*, and *Pseudomonas aeruginosa*) were determined. Compounds substituted with amino or 8-quinolinyl moieties exhibited enhanced antimicrobial activity with MIC values ranging between 6.25 and 12.5 µg/mL.

Karegoudar et al. [54] successfully developed a series of 4-substituted 2-(2,3,5-trichlorophenyl)-1,3-thiazoles (compounds **12** and **13**, Figure 5) and 4-substituted 2-(2,3,5-trichlorophenylidenehydrazino)-1,3-thiazoles (compound **14**, Figure 5) and evaluated their antimicrobial activity on four bacterial strains and four fungal strains. The best antibacterial activity was observed among derivatives substituted with 4-(methylthio)phenyl, salicylamide, *N*-methylpiperazine, or 4,6-dimethyl-2-mercaptopyrimidine residues, and the best antifungal activity was reported among compounds substituted with 3-pyridyl, biphenyl, or 4-mercaptopyrazolopyrimidine moieties.

In a study by Lino et al. [55], a novel series of thiazole derivatives bearing a hydrazone group was synthesized. The biological activity of the thiosemicarbazone intermediates and target compounds was evaluated in vitro against seven fungal strains: *Candida albicans, C. krusei, C. parapsilosis, C. tropicalis, Cryptococcus neoformans, C. gatti*, and *Paracoccidioides brasiliensis*. It was noted that the thiazole nucleus was essential for antifungal activity, since the activity of the intermediate compounds was absent. Furthermore, the presence of a hydrophobic aliphatic chain linked to the hydrazone functional group and the chlorine atom in the *para* position on the benzene ring were associated with an increase in antifungal activity. Compound **15** (Figure 5) showed superior activity compared to fluconazole on all *Candida* and *Cryptococcus* species used in this assay.

Also, ongoing research is focused on the development of new antimicrobial compounds with a different mechanism of action than those currently used in therapy. FabH, also known as β-ketoacyl-(acyl-carrier-protein)synthase III (KAS III), is a key enzyme involved in the biosynthesis of bacterial fatty acids, being responsible for the first condensation reaction between acetyl-coA and malonyl–acyl carrier protein (ACP), with the formation of acetoacetyl-ACP. FabH is found in many bacterial pathogens including *Escherichia coli, Staphylococcus aureus, Mycobacterium tuberculosis, Enterococcus faecium, Streptococcus pneumoniae, Pseudomonas aeruginosa, Neisseria meningitidis*, and *Haemophilus influenzae*, most of them possessing high levels of resistance to currently authorized antimicrobial drugs [56]. The three-dimensional structure of the FabH protein is well preserved among many Gram-positive and Gram-negative bacteria. Consequently, molecules with a FabH inhibitory effect could have a broad-spectrum antimicrobial activity [57].

Cheng et al. [58] reported the synthesis of a new series of 2-phenylacetamido-thiazole derivatives with potent *Escherichia coli* KAS III (ecKAS III) inhibitory activity. The antibacterial screening was performed on two Gram-negative bacterial strains (*Escherichia coli* and *Pseudomonas aeruginosa*) and two Gram-positive bacterial strains (*Bacillus subtilis* and *Staphylococcus aureus).* The most active derivative (compound **16**, Figure 5) showed favorable MIC values, ranging between 1.56 and 6.25 μg/mL, on all tested bacterial strains. It also exhibited the highest ecKAS III inhibitory activity, with an IC_50_ value of 5.3 μM.

Lv et al. [59] synthesized a new series of thiazole Schiff bases and evaluated their antibacterial activity against several Gram-positive and Gram-negative bacterial strains (*Bacillus subtilis*, *Staphylococcus aureus*, *Streptococcus faecalis*, *Escherichia coli*, *Pseudomonas aeruginosa*, and *Enterobacter cloacae*). The MIC values of the tested compounds were compared to those of penicillin G and kanamycin B, used as reference substances. Compounds **17** and **18** (Figure 5) showed a better antibacterial effect compared to kanamycin B against *E. coli* and a favorable activity against other bacterial strains, thus indicating the presence of broad-spectrum antibacterial activity. Moreover, the two derivatives possessed good in vitro ecKAS III inhibitory activity, with IC_50_ values equal to 3.6 and 6.8 μM, respectively.

### 4.2. Antiprotozoal Activity

**Figure 6:**
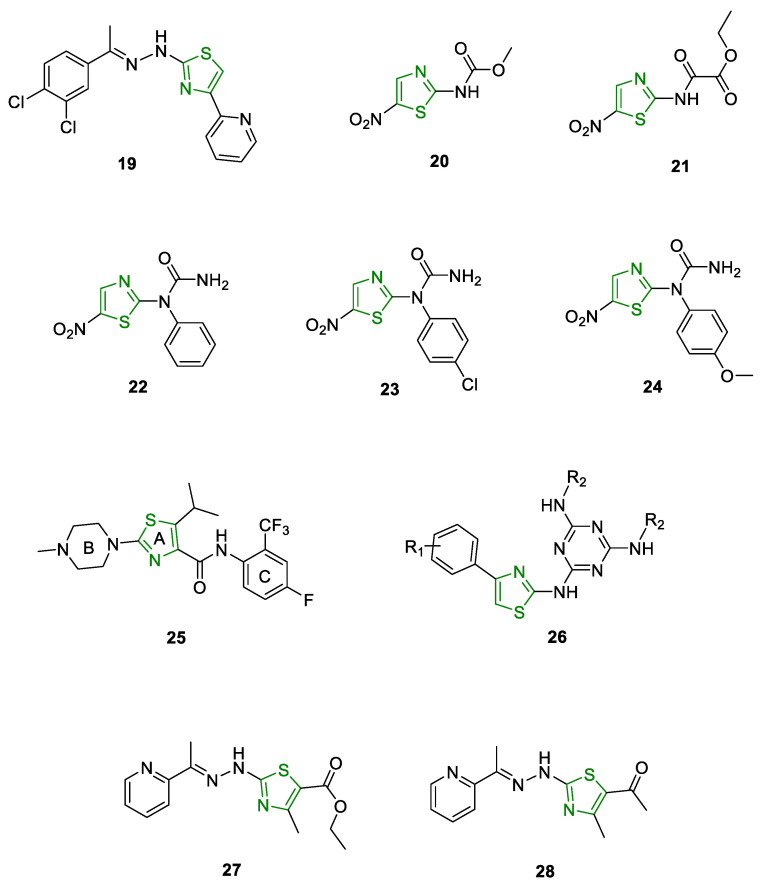
Thiazole derivatives with antiprotozoal activity.

Protozoan infections are very common in some regions throughout the world, being endemic in less developed countries. The development of novel molecules with antiprotozoal activity is necessary, given the small number of antiprotozoal drugs currently on the market, their relatively low efficacy and high toxicity, as well as the spread of resistance [60].

de Oliveira Filho et al. [61] synthesized 22 novel thiazole derivatives and investigated their anti-*T. cruzi* activity. Compound **19** (Figure 6) proved to be very potent against trypomastigotes of the Y strain, with an IC_50_ value equal to 0.37 µM, being nearly 28 times more active than benznidazole, used as a reference drug. It also reduced blood parasitemia in mice. Moreover, the new compounds possessed a good drug-likeness profile.

A novel series of 2-acylamino-5-nitro-1,3-thiazoles was synthesized by Nava-Zuazo et al. [62] and evaluated against the following protozoa: *Giardia intestinalis, Trichomonas vaginalis, Leishmania amazonensis*, and *Trypanosoma cruzi.* Methylcarbamate derivative (compound **20**, Figure 6) displayed the highest giardicidal activity, with an IC_50_ value equal to 10 nM, being more potent than metronidazole and nitazoxanide, used as reference drugs. Ethyloxamate derivative (compound **21**, Figure 6) revealed the highest trichomonicidal effect. Ureic derivatives (compounds **22**, **23**, and **24**, Figure 6) showed moderate antileishmanial activity. None of the new synthesized 2-acylamino-5-nitro-1,3-thiazoles showed trypanocidal effect at a concentration under 50 µM.

Bueno et al. [63] synthesized and investigated the antimalarial activity of new thiazole derivatives. The structure–activity relationship study indicated that the activity of the obtained compounds was not significantly influenced by pharmacomodulations of the B ring if a nitrogen atom from a piperidine, piperazine, or *N*-methylpiperazine moiety was directly linked at position 2 of the thiazole ring (compound **25**, Figure 6). At the same time, it was observed that the structural integrity of the B ring was, therefore, essential for the biological activity, since its opening led to a significant decrease in the antimalarial activity. The presence of an ethyl or isopropyl radical at position 5 of the thiazole nucleus was favorable for the activity. Moreover, substitutions of the C ring greatly influenced the antimalarial activity. The *ortho* monosubstitution with non-voluminous electron-withdrawing groups was essential for the activity. Substitution at both *ortho* positions led to inactive compounds. If the *para* position is occupied, the substituent must be a small-volume atom such as fluorine.

Sahu et al. [64] synthesized 20 thiazole-1,3,5-triazine hybrids as potential antimalarial agents (compound **26**, Figure 6). Two strains of *P. falciparum*, one sensitive (3D-7) and one resistant (Dd-2) to chloroquine, were employed for this assay. All compounds proved to be active against the chloroquine-sensitive strain, with IC_50_ values ranging between 10.03 and 54.58 µg/mL. Among them, eight thiazole-1,3,5-triazine derivatives displayed IC_50_ values from 11.29 to 40.92 µg/mL against the chloroquine-resistant strain.

Makam et al. [65] developed a series of 2-(2-hydrazinyl)thiazoles and evaluated the antimalarial activity of the new compounds against *Plasmodium falciparum.* The compounds contain various substituents at positions 2, 4, and 5, including phenyl, pyridyl, and five-membered rings. Thus, it was observed that the presence of a 2-pyridyl hydrazinyl residue at position 2 of the thiazole nucleus was associated with enhanced antimalarial activity. Moreover, compounds **27** and **28** (Figure 6) substituted at position 5 with a -COOC_2_H_5_ and a -COCH_3_ group, respectively, showed the highest inhibitory activity, with IC_50_ values equal to 0.725 and 0.648 µM, respectively. The enhanced biological activity owing to the presence of a carbonyl group at position 5 could be explained due to the keto-enol tautomerism that could influence the pharmacological properties.

### 4.3. Antitumor Activity

A major problem of cancer therapy remains the lack of selectivity of currently available antitumor agents, and consequently their high toxicity, and the development of the cancer cell resistance phenomenon. Nowadays, research in the field is focused on targeted therapy, numerous compounds that act specifically on a particular molecular structure being synthesized [66].

Thiazole derivatives have demonstrated remarkable anticancer potential due to the high affinity toward various biological targets involved in cancer pathogenesis, such as PRL-3, SHP-2, and JSP-1 phosphatases; tumor necrosis factor (TNFα); antiapoptotic biocomplex Bcl-XL-BH3; integrin avb3; and protein kinases Pl3K, CDK, LIM, EGFR, and VEGFR [66,67,68].

**Figure 7:**
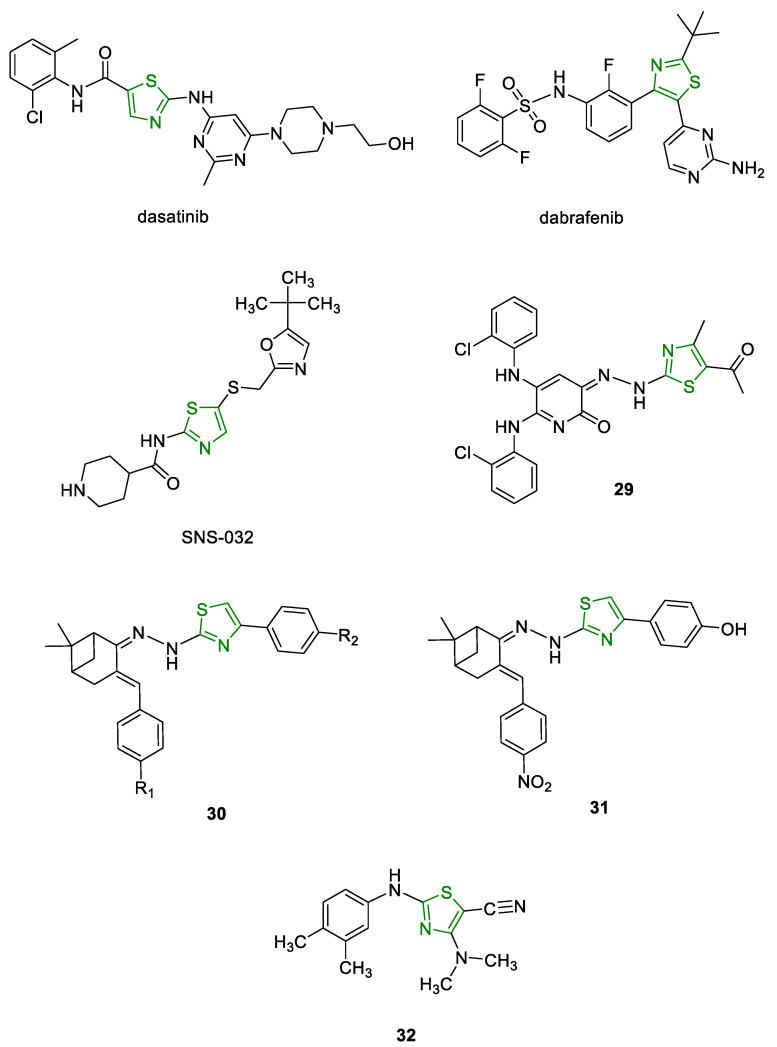
Thiazole derivatives with antitumor activity.

Kinase-mediated signaling pathways are involved in tumor proliferation and survival, as well as in evasion of the host immune system response [69]. Two novel thiazole-based compounds with tyrosine kinase inhibitory activity, dasatinib and dabrafenib (Figure 7), are currently authorized for clinical use [70].

Dasatinib is approved for treatment of children and adults who are diagnosed with Philadelphia chromosome-positive chronic myeloid leukemia or Philadelphia chromosome-positive acute lymphoblastic leukemia. It is a multitarget inhibitor that inhibits BCR-ABL (breakpoint cluster region-Abelson) and other tyrosine kinases [71]. Dabrafenib is a cancer medicine used to treat adult patients with melanoma or metastatic non-small cell lung cancer with BRAF V600E mutation. The mechanism of action consists of the inhibition of BRAF kinase activity involved in stimulating cell division [72,73]. Moreover, a potent and selective inhibitor of cyclin-dependent kinases Cdk2, Cdk7, and Cdk9, the thiazole derivative SNS-032 is in phase I clinical trials [66].

Also, the antitumor potential of thiazole derivatives is highlighted by the large number of compounds that have been shown to possess in vitro cytotoxic activity [74]

Xie et al. [75] successfully synthesized a series of hybrid molecules of thiazole combined with a 2-pyridone ring. The antiproliferative activity of the new compounds was assessed in vitro by the MTT (3-(4,5-dimethylthiazol-2-yl)-2,5-diphenyltetrazolium bromide) method against three human tumor cell lines: colon cancer HTC-116 cells, gastric carcinoma MGC803 cells, and liver cancer Huh7 cells. The results were compared with those of 5-fluorouracil, used as a reference substance. The most active compound (**29**, Figure 7) showed IC_50_ values equal to 3.15 ± 1.68 µM for the MGC803 cell line and 8.17 ± 1.89 µM for the HTC-116 cell line, lower than the obtained IC_50_ values of 5-fluorouracil (25.54 ± 0.05 and 11.29 ± 1.06 µM, respectively). It was also observed that the presence of halogen atoms (Cl, Br, F) on the benzene rings led to an increase in inhibitory activity, while the presence of electron-donating groups (-CH_3_, -OCH_3_) led to a decrease in antiproliferative activity.

Wang et al. [76] developed a number of thiazole derivatives containing a β-pinene residue (compound **30**, Figure 7). The antiproliferative activity of these compounds was evaluated against three tumor cell lines: murine colorectal carcinoma CT-26 cells, human cervical carcinoma HeLa cells, and human hepatocellular carcinoma SMMC-7721 cells. The best inhibitory activity against the tested tumor cell lines was present when a strong electron-donating group (-OH) was introduced into the R_2_ position. In addition, the most active compound (**31**, Figure 7) was substituted with a strong electron-withdrawing group (-NO_2_) at the R_1_ position. It exhibited a strong antiproliferative effect, with IC_50_ values ranging between 3.48 and 8.84 µM, the highest inhibitory activity being recorded against the HeLa cell line.

Anuradha et al. [77] synthesized a series of thiazole derivatives as potential antitumor agents. Their inhibitory activity was evaluated in vitro against two tumor cell lines (Bcl-2 Jurkat and A-431 cell lines) and a normal cell line (ARPE-19 cells), using doxorubicin as a reference substance. IC_50_ values of the most active compound (**32**, Figure 7) were lower than those of doxorubicin (34.77 μM in the Bcl-2 Jurkat cell line compared to 45.87 μM in the case of doxorubicin and 34.31 μM in the A-431 cell line compared to 42.37 μM in the case of doxorubicin). The absence of toxicity in the normal cell line was also observed. In silico studies and experimental results showed that this compound induced apoptosis in tumor cells by binding to Bcl-2 protein.

## 5. Synthesis of Bisthiazole Derivatives (Thiazolyl-Thiazoles and Thiazolyl-Linker-Thiazoles)

### 5.1. Synthesis of Thiazolyl-Thiazole Derivatives

**Scheme 15:**
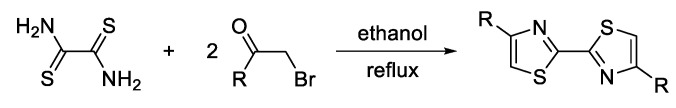
Synthesis of 2,2′-bisthiazole derivatives starting from dithiooxamide.

The synthesis of symmetrical 2,2′-bisthiazole compounds occurs most frequently by condensation of dithiooxamide with α-bromoketones at a ratio of 1:2 in ethanol at reflux (Scheme 15) [78,79,80].

**Scheme 16:**
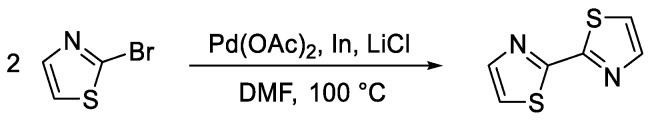
Synthesis of 2,2′-bisthiazole starting from 2-bromothiazole.

Kooyeon Lee and Phil Ho Lee [81] reported a method for the synthesis of 2,2′-bisthiazole by a homocoupling reaction of 2-bromothiazole in the presence of Pd(OAc)_2_, indium, and LiCl, in DMF, at a temperature of 100 °C (Scheme 16).

**Scheme 17:**
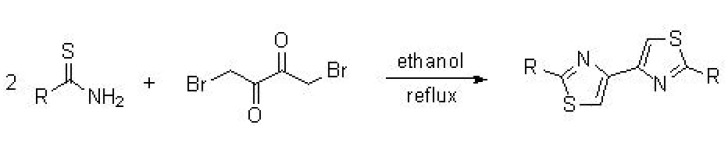
Synthesis of 4,4′-bisthiazole derivatives starting from thioamides.

The synthesis of symmetrical 4,4′-bisthiazole derivatives is most often performed by a condensation reaction of thioamides with 1,4-dibromo-2,3-butanedione, in a molar ratio of 2:1, in ethanol at reflux (Scheme 17) [82,83].

**Scheme 18:**
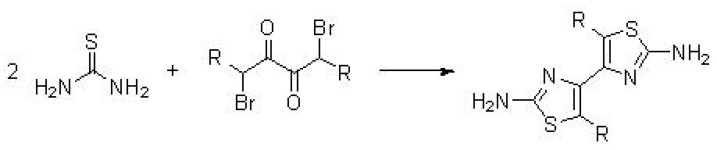
Synthesis of 4,4′-bis(2-aminothiazole) derivatives starting from thiourea.

By condensation of two equivalents of thiourea with an equivalent of 1,4-dibromo-2,3-butanedione or 2,5-dibromo-3,4-hexandione, 4,4′-bis(2-aminothiazole) derivatives were obtained (Scheme 18) [84,85]. 

**Scheme 19:**
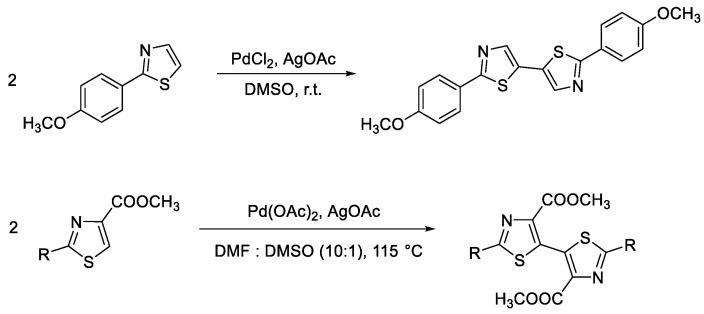
Synthesis of 5,5-bisthiazole derivatives by the homocoupling reaction.

Several methods for the synthesis of 5,5′-bisthiazole compounds, starting from halogenated derivatives by transition-metal-catalyzed arylation or cross-coupling reactions, have been reported in the literature [86,87]. Also, the homocoupling reaction of 2-(4-methoxyphenyl)thiazole and thiazole-4-carboxylate substrates, in the presence of Pd(II) salts as a catalyst, led to symmetrical 5,5′-bisthiazoles (Scheme 19) [88,89].

**Scheme 20:**
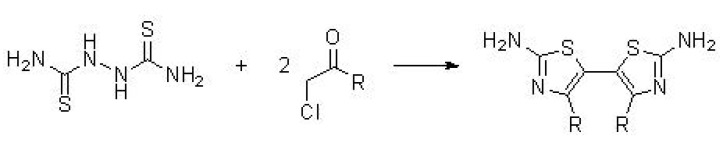
Synthesis of 5,5′-bis(2-aminothiazole) derivatives.

Boong Won Lee and Seung Dal Lee [85] reported the synthesis of a novel series of 5,5′-bis(2-aminothiazole) derivatives by the condensation of 2,5-dithiobiurea with α-halocarbonyl compounds in a ratio of 1:2 (Scheme 20).

**Scheme 21:**
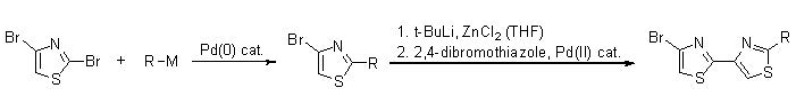
Synthesis of 2,4′-bisthiazole derivatives.

Bach and Heuser [90] synthesized 2,4′-bisthiazole derivatives starting from 2,4-dibromothiazole, according to the reactions illustrated in Scheme 21. Initially, a palladium-catalyzed cross-coupling reaction occurred between 2,4-dibromothiazole and an organometallic compound R-M. A new carbon–carbon bond was formed at position 2 of the thiazole ring, resulting in the synthesis of 2-substituted 4-bromothiazole derivatives. Subsequently, in the presence of *tert*-butyllithium, a bromine–lithium exchange took place. Finally, by the cross-coupling reaction with a new molecule of 2,4-dibromothiazole, 2,4′-bisthiazole derivatives were obtained.

**Scheme 22:**
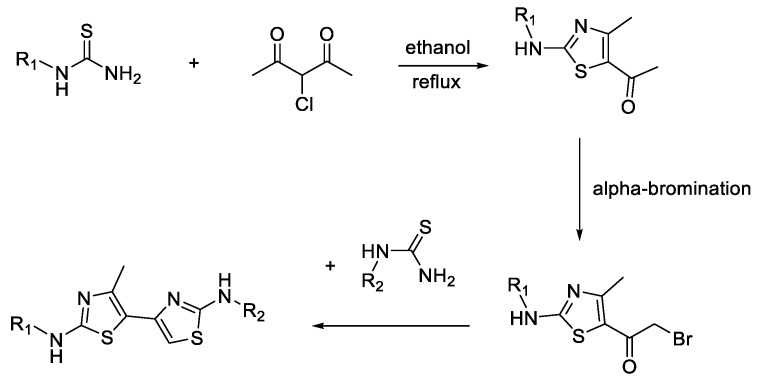
Synthesis of 4,5′-bisthiazole derivatives.

The synthesis of 4,5′-bisthiazole compounds was performed in several steps, starting from different thiourea derivatives (Scheme 22). First, a condensation reaction between various thioureas and 3-chloro-2,4-pentanedione was performed by refluxing in ethanol, giving the corresponding thiazole derivatives. Subsequently, the acetyl group was alpha-brominated with bromine in acetic acid, pyridinium tribromide, or phenyltrimethylammonium tribromide. Last, the condensation of alpha-bromoketone bearing a thiazole scaffold with various thioureas afforded the desired 4,5′-bisthiazole compounds [91,92,93]. 

### 5.2. Synthesis of Thiazolyl-Linker-Thiazole Compounds

Numerous protocols for the synthesis of thiazolyl-linker-thiazole compounds have been reported in the literature, most of them being based on the Hantzsch condensation reaction. The reaction can occur in a single step, when the two thiazole rings are formed simultaneously, using starting compounds such as bis-thiosemicarbazones, 2,5-dithiobiurea, bis-hydrazonoyl halides, or dihalo diketones, or in two steps when the two thiazole rings are formed successively [94,95,96,97,98,99].

**Scheme 23:**
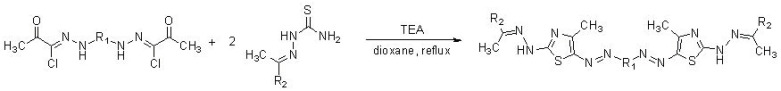
Synthesis of bisthiazole derivatives starting from bis-hydrazonoyl chlorides.

Gomha et al. [100] synthesized bisthiazole derivatives by the condensation reaction between various thiosemicarbazones and bis-hydrazonoyl chlorides, in the presence of triethylamine, under reflux conditions in dioxane, for 4–8 h (Scheme 23). 

**Scheme 24:**
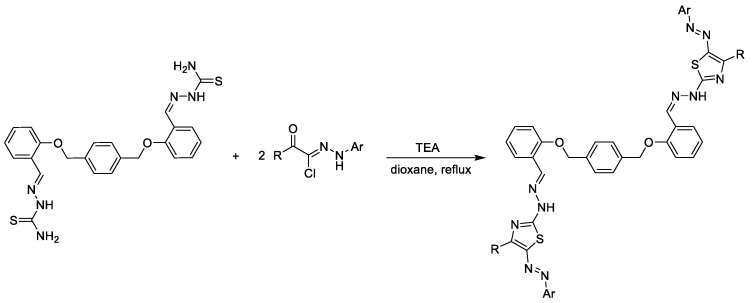
Synthesis of bisthiazole derivatives starting from bis-thiosemicarbazones.

Mahmoud et al. [101] developed a new synthetic method starting from a bis-thiosemicarbazone derivative, which contains a voluminous linkage. The compound reacted with two equivalents of various hydrazonoyl chlorides, in the presence of triethylamine, in refluxing dioxane or α-halocarbonyl derivatives, in refluxing ethanol, leading to the formation of novel bisthiazole derivatives (Scheme 24).

## 6. Biological Activity of Bisthiazole Derivatives

### 6.1. Antimicrobial Activity

Bisthiazoles are present in the structure of several natural compounds with antimicrobial properties. Moreover, synthetic bisthiazole derivatives have been developed as potential antibacterial or antifungal agents.

**Figure 8:**
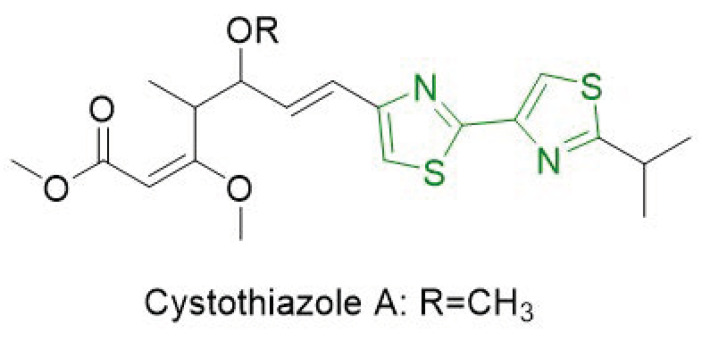
Chemical structure of cystothiazoles.

Cystothiazoles (Figure 8) were isolated in 1998 by Sakagami and co-workers from *Cystobacter fuscus* and demonstrated good antifungal activity against *Phytophthora capsici* strain (0.05–5 µg/disk) [102]. In addition, cystothiazole A was active against many fungal strains, including *Candida albicans* (MIC 0.4 μg/mL), but inactive against the tested bacterial strains [103].

**Figure 9:**
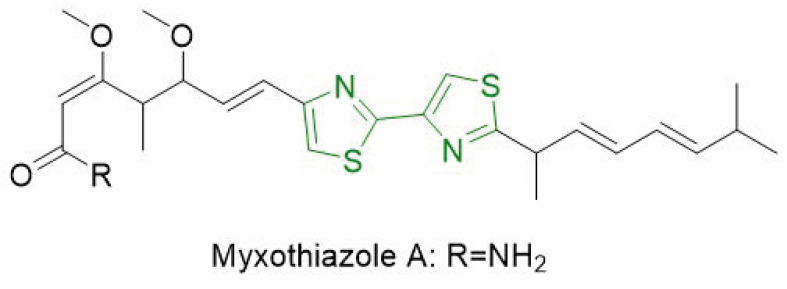
Chemical structure of myxothiazoles.

Myxothiazoles (Figure 9), isolated from *Myxococcus fulvus* cultures, showed moderate antifungal activity against the *Candida albicans* strain [104]. Comparing cystothiazole A with myxothiazole A, it could be observed that the former was more active and less cytotoxic [103]. 

**Figure 10:**
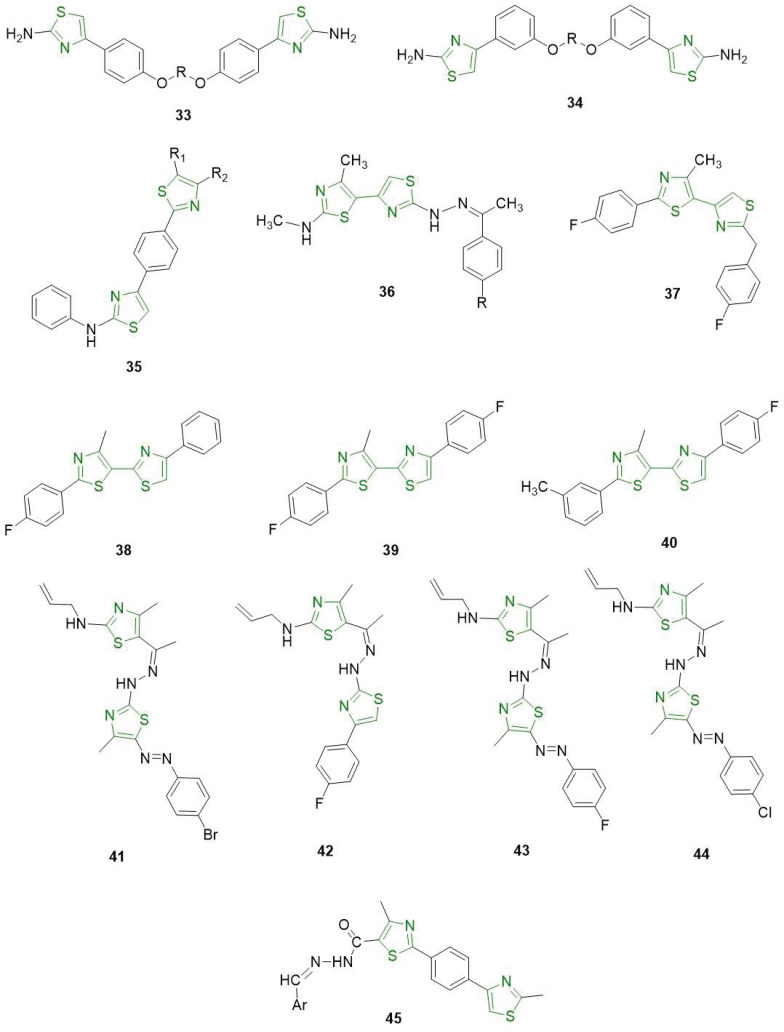
Bisthiazole derivatives with antimicrobial activity.

Mahmoodi et al. [105] synthesized a novel series of bisthiazoles by refluxing bis-2-bromo-acetophenones and thiourea, in anhydrous ethanol, for 6 h (compounds **33** and **34**, Figure 10). Their antibacterial effect was evaluated against four bacterial strains (*Pseudomonas aeruginosa, Escherichia coli, Micrococcus luteus*, and *Bacillus subtilis*). Tetracycline was used as a reference substance. Gram-positive bacteria were more susceptible to the action of the synthesized compounds than Gram-negative bacteria. The MIC values of the tested compounds were lower than those of tetracycline against Gram-positive bacteria. In addition, increased antibacterial activity was observed in the case of compounds with higher lipophilicity.

Bikobo et al. [106] synthesized a series of 1,4-phenylenebisthiazole compounds (**35**, Figure 10) and investigated their antimicrobial activity against three bacterial strains (*Enterococcus faecalis, Staphylococcus aureus*, and *Salmonella typhimurium*) and two fungal strains (*Candida albicans* and *Candida krusei*). The most active compound in this series was the 1,4-phenylenebisthiazole derivative substituted with a 3-carbamoyl-4-hydroxyphenyl residue.

Althagafi et al. [107] synthesized a series of 4,5′-bisthiazole compounds (**36**, Figure 10) and performed an antimicrobial screening using four Gram-positive bacterial strains (*Staphylococcus aureus, Staphylococcus epidermidis, Bacillus subtilis*, and *Streptococcus pyogenes*), four Gram-negative bacterial strain (*Pseudomonas aeruginosa, Escherichia coli, Klebsiella pneumoniae*, and *Salmonella typhimurium*), and two fungal strains (*Aspergillus niger* and *Geotrichum candidum*). The activity was evaluated by measuring the diameter of the inhibition zone, and the results were compared to those of ampicillin, gentamicin, and amphotericin B, used as reference substances. Most of the compounds exhibited a superior inhibitory effect against the *Aspergillus niger* strain than amphotericin B. They also displayed moderate to good antibacterial activity, several derivatives being more active on some bacterial strains than the reference substances. Still, the tested compounds had no activity against *Streptococcus pyogenes* and *Pseudomonas aeruginosa* strains.

Abhale et al. [108] synthesized a series of 4′,5-bisthiazole derivatives and tested their antimicrobial activity against four bacterial strains (*Bacillus subtilis, Staphylococcus aureus, Escherichia coli*, and *Proteus vulgaris)* and one mycobacterial strain (*Mycobacterium smegmatis*). Amoxicillin, ciprofloxacin, rifampicin, and isoniazid were used as reference substances for antimicrobial activity assays. The most active compound (**37**, Figure 10) showed superior efficacy than isoniazid against *M. smegmatis* (MIC 30.38 µg/mL) and superior activity than amoxicillin and ciprofloxacin against *B. subtilis*, *E. coli*, and *P. vulgaris*. 

Based on the results obtained for 4′,5-bisthiazole derivatives, a new series of 2,5′-bisthiazole compounds was synthesized and evaluated against two mycobacterial strains (*M. tuberculosis* H37Ra and *M. bovis* BCG). Compounds **38**, **39**, and **40** (Figure 10) displayed good antimycobacterial activity against both strains, with MIC_90_ and IC_50_ values ranging between 9.64 and 23.64 µg/mL and between 0.82 and 4.55 µg/mL, respectively. Subsequently, 2,5′-bisthiazole derivatives were subjected to an antibacterial and antifungal screening against two Gram-negative bacterial strains (*Escherichia coli* and *Pseudomonas fluorescence*), two Gram-positive bacterial strains (*Staphylococcus aureus* and *Bacillus subtilis*), and a fungal strain (*Candida albicans*), exhibiting moderate to good antibacterial activity and moderate antifungal activity [109].

Bondock and Fouda [110] synthesized a series of bisthiazolyl hydrazones and investigated their antimicrobial activity against Gram-positive and Gram-negative bacteria and fungi. Regarding the inhibitory activity against Gram-positive bacteria, compound **41** (Figure 10) was twice as active as ampicillin against *S. pneumoniae* (MIC 0.06 µg/mL) and compound **42** (Figure 10) was four times more active than ampicillin against *S. pneumoniae* (MIC 0.03 µg/mL) and *B. subtilis* (MIC 0.06 µg/mL). Regarding the inhibitory activity against Gram-negative bacteria, compounds **43** and **44** (Figure 10) showed a gentamicin-like activity against *K. pneumoniae* (MIC 0.03 µg/mL). The results of the antifungal activity assay showed that compound **43** (Figure 10) was four times more potent against *A. fumigatus* (MIC 0.03 µg/mL) compared to the activity of the reference substance, amphotericin B (MIC 0.12 µg/mL). In addition, compound **41** (Figure 10) showed a similar activity to that of amphotericin B against this fungal strain (MIC 0.12 µg/mL). In this study, it was observed that the presence of two thiazole moieties merged through a hydrazone group is associated with increased antibacterial and antifungal activity.

A novel series of acylhydrazones containing a 1,4-phenylenebisthiazole nucleus (compound **45**, Figure 10) were synthesized by Borcea et al. [111]. Their anti-*Candida* activity was evaluated in vitro against two strains of *Candida albicans* and two non-albicans strains (*C. parapsilosis* and *C. krusei*). Several compounds have proved to be as active as fluconazole, used as a reference drug. An in silico molecular docking study was performed in order to investigate the binding mode of the tested compounds toward lanosterol 14α-demethylase. The results showed that all interactions between the tested compounds and lanosterol 14α-demethylase involved amino acids residues from the access channel, without interaction with the heme-iron catalytic site.

### 6.2. Antiprotozoal Activity

Liu et al. [112] developed new DB766 analogues in which the terminal pyridyl nucleus was replaced with various heterocyclic rings, including the thiazole nucleus. The biological activity of the new compounds was evaluated against *Trypanosoma cruzi*, *Trypanosoma brucei rhodesiense*, *Leishmania amazonensis*, and *Plasmodium falciparum*. Among the bis-arylimidamide thiazole derivatives obtained, compounds **46**, **47**, and **48** exhibited IC_50_ values between 0.17 and 0.3 µM against *L. amazonensis* and compound **48** showed promising activity against *T. brucei rhodesiense,* with an IC_50_ value equal to 12 nM. Moreover, five of the bis-arylimidamide thiazole derivatives showed favorable activity against *P. falciparum*, with IC_50_ values ranging between 9 and 86 nM. None of the bis-arylimidamide thiazoles showed anti-*T. cruzi* activity under 1 µM.

**Figure 11:**
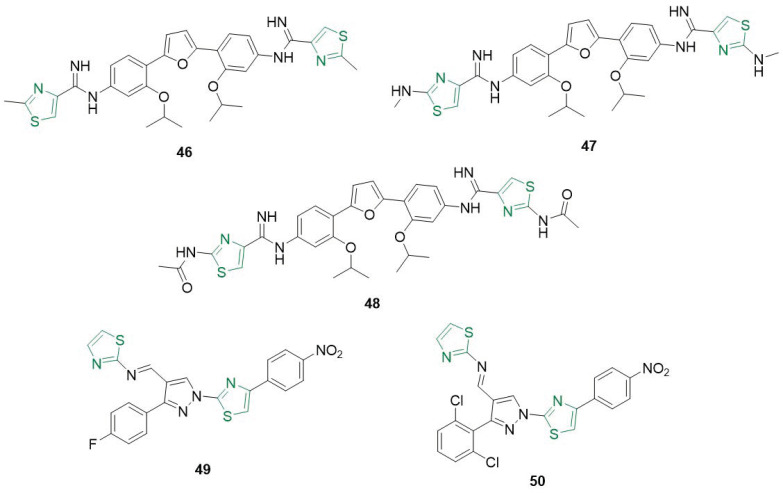
Bisthiazole derivatives with antiprotozoal activity.

Bansal et al. [113] synthesized several bisthiazole derivatives containing a pyrazole moiety and investigated their antimalarial activity against *Plasmodium falciparum*. Compounds **49** and **50** (Figure 11) with 4-fluoro and 2,6-dichloro substitution, respectively, exhibited excellent activity against *P. falciparum*, with IC_50_ values equal to 0.23 and 0.31 µg/mL, respectively. Furthermore, it was noted that the presence of an electron-withdrawing group such as 4-F and 2,4-*di*Cl was associated with an enhanced antimalarial effect, while the presence of an electron-donating group such as 4-CH_3_ led to a decrease in antimalarial activity.

### 6.3. Antitumor Activity

**Figure 12:**
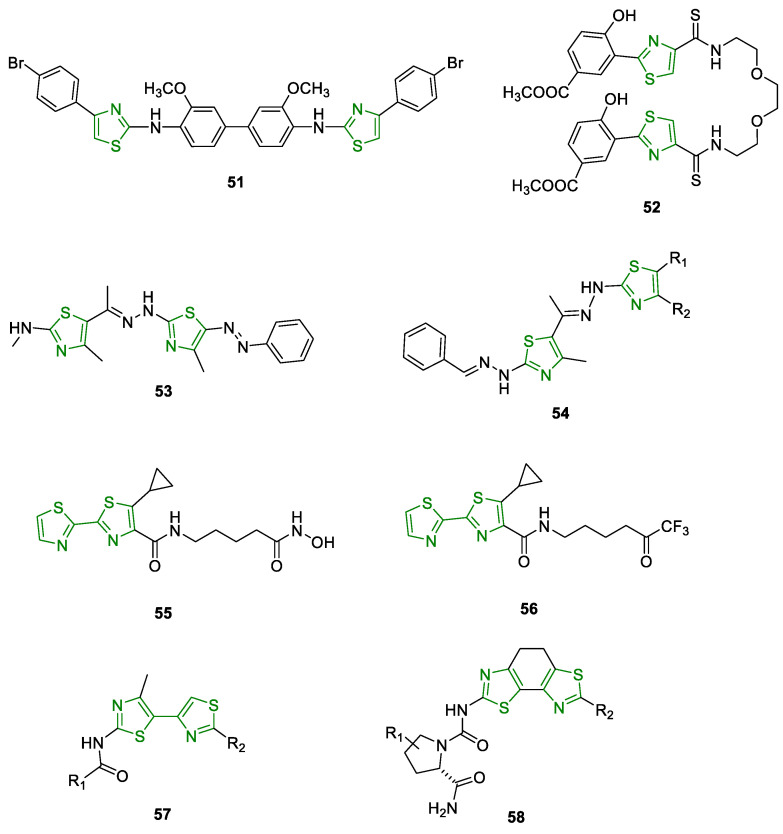
Bisthiazole derivatives with antiproliferative activity.

Turan-Zitouni et al. [114] synthesized a series of new bisthiazole derivatives and evaluated their antiproliferative activity against three tumor cell lines (A549, C6, and 5RP7 H-ras), as well as their cytotoxic effect against the murine embryonic fibroblast cell line NIH/3T3. The best activity was recorded for the *p*-bromo substituted bisthiazole derivative (compound **51**, Figure 12) against the rat glioma C6 cell line, with an IC_50_ value (11.3 ± 1.2 μg/mL) being similar to that of mitoxantrone (11.0 ± 1.7 μg/mL), used as a reference substance. It also exhibited a weak cytotoxic effect on the normal cell line NIH/3T3.

Rodriguez-Lucena et al. [115] developed a series of bis-2-(2-hydroxyphenyl)-thiazole-4-carboxamides and bis-2-(2-hydroxyphenyl)-thiazole-4-thiocarboxamides as iron chelators. The antiproliferative activity of the compounds was evaluated in vitro against 13 human tumor cell lines (KB, HCT116, HT29, HCT15, MCF7, MCF7R, SK-OV-3, HepG2, PC-3, A549, HL60, K562, and SF268) representative of different types of carcinoma. The results were compared with those of deferoxamine. The most active compound, the dithioamide derivative **52** (Figure 12), showed a superior antiproliferative effect of deferoxamine on almost all tested cell lines. Its inhibitory activity was highlighted on the lung carcinoma A549 cell line, with 98% inhibition of cell proliferation at 10 μM.

Farghaly et al. [116] synthesized a novel series of bisthiazole derivatives by reacting the thiazole thiosemicarbazone key intermediate with alpha-haloketones, in refluxing dioxane, containing a catalytic amount of triethylamine. Their antitumor activity was evaluated in vitro against colon (HCT-116) and hepatocellular (HepG2) cancer cell lines. The most active compound (**53**, Figure 12) showed a good cytotoxic effect against HCT-116 and HepG2 cell lines, with IC_50_ values equal to 6.6 and 4.9 μg/mL, respectively. 

Sayed et al. [117] developed novel thiazolyl-hydrazono-ethylthiazole derivatives (**54**, Figure 12) as potential anticancer agents. The biological assay was carried out using three human tumor cell lines: colon carcinoma HCT-116, the more resistant human colorectal cancer HT-29, and hepatocellular carcinoma HepG2 cell lines. The results showed promising cytotoxic activity of several compounds. Moreover, it was observed that the presence of an electron-withdrawing substituent, such as chlorine or bromine, was associated with increased inhibitory activity, while the presence of an electron-donating group (-CH_3_, -OCH_3_) led to a decrease in cytotoxic activity. The study of the mechanism of action in HCT-116 cells revealed that apoptosis occurred through the Bcl-2 family.

Chen et al. [118] developed a number of histone deacetylase (HDAC) inhibitors, based on the structure of a natural HDAC inhibitor, largazole, and clinically approved HDAC inhibitors, vorinostat and panobinostat. Thus, a series of 2,2′-bisthiazoles containing a hydroxamic acid group were synthesized. In vitro studies showed that the bisthiazole derivative **55** (Figure 12) inhibited the enzymatic activity of HDAC, leading to an increase in the acetylation levels of histones H3 and H4 and apoptosis of T lymphocytes in a dose-dependent manner.

Subsequently, Gong et al. [119] synthesized a new series of 2,2′-bisthiazole compounds by replacing the hydroxamic acid residue with other functional groups such as *N*-hydroxyurea, *o*-diaminobenzene, trifluoromethyloxadiazole or trifluoromethyl ketone. Compound **56** (Figure 12), which contains the trifluoromethyl ketone residue, exhibited the best inhibitory activity against HDAC 1, 3, 4, and 6, with IC_50_ values between 20.81 and 31.54 nM. It also showed very good antiproliferative activity against multiple myeloma (MM.1S, RPMI 8226, NCI-H929, and LP1) and lymphoma (Mino and JeKo-1) cell lines.

Fairhurst et al. [120] synthesized a novel series of selective phosphatidylinositol 3-kinase alpha (PI3Kα) inhibitors with a 4,5-bisthiazole scaffold (compound **57**, Figure 12). The best PI3Kα inhibitory activity was recorded for compounds in which the amide group was substituted with a *cis*-3-methylproline amide residue and position 2 of the thiazole ring was substituted with a quaternary alkyl residue. Subsequently, based on the obtained results, a new series of 4,5-dihydrobenzo[1,2-*d*:3,4-*d*]bisthiazole tricyclic compounds (**58**, Figure 12) was developed, the enzyme inhibition ability of these molecules being superior to 4,5-bisthiazoles [121].

## 7. Conclusions

Thiazole is an important scaffold in medicinal chemistry, easy to synthesize from a wide variety of starting compounds. To date, many organic compounds containing one or more thiazole rings, showing promising antimicrobial, antiprotozoal, and antitumor activities, have been added to the literature. The detailed knowledge of various synthetic strategies and biological effects would be of great help in the drug discovery and development process.

## Data Availability

No new data were created or analyzed in this study. Data sharing is not applicable to this article.
